# Multiple Respiratory Syncytial Virus Introductions Into a Neonatal Intensive Care Unit

**DOI:** 10.1093/jpids/piaa026

**Published:** 2021-03-26

**Authors:** Erica Billig Rose, Erica J. Washington, Lijuan Wang, Isaac Benowitz, Natalie J. Thornburg, Susan I. Gerber, Teresa C. T. Peret, Gayle E. Langley

**Affiliations:** 1 Division of Viral Diseases, National Center for Immunization and Respiratory Diseases, Centers for Disease Control and Prevention, Atlanta, Georgia, USA;; 2 Epidemic Intelligence Service, Centers for Disease Control and Prevention, Atlanta, Georgia, USA;; 3 Louisiana Department of Health, Office of Public Health, Infectious Disease Epidemiology Section, New Orleans, Louisiana, USA;; 4 IHRC Inc, contracting agency to the Division of Viral Diseases, National Center for Immunization and Respiratory Diseases, Centers for Disease Control and Prevention, Atlanta, Georgia, USA;; 5 Division of Healthcare Quality Promotion, National Center for Emerging and Zoonotic Infectious Diseases, Centers for Disease Control and Prevention, Atlanta, Georgia, USA

**Keywords:** respiratory syncytial virus, RSV, RSV outbreak

## Abstract

**Background.:**

Outbreaks of respiratory syncytial virus (RSV) in neonatal intensive care units (NICUs) are of concern because of the risk of severe disease in young infants. We describe an outbreak of RSV in a NICU and use whole genome sequencing (WGS) to better understand the relatedness of viruses among patients.

**Methods.:**

An investigation was conducted to identify patients and describe their clinical course. Infection control measures were implemented to prevent further spread. Respiratory specimens from outbreak-related patients and the community were tested using WGS. Phylogenetic trees were constructed to understand relatedness of the viruses.

**Results.:**

Seven patients developed respiratory symptoms within an 11-day span in December 2017 and were diagnosed with RSV; 6 patients (86%) were preterm and 1 had chronic lung disease. Three patients required additional respiratory support after symptom onset, and none died. Six of 7 patients were part of the same cluster based on > 99.99% nucleotide agreement with each other and 3 unique single-nucleotide polymorphisms were identified in viruses sequenced from those patients. The seventh patient was admitted from the community with respiratory symptoms and had a genetically distinct virus that was not related to the other 6. Implementation of enhanced infection control measures likely limited the spread.

**Conclusions.:**

Using WGS, we found 2 distinct introductions of RSV into a NICU, highlighting the risk of healthcare-associated infections during RSV season. Early recognition and infection control measures likely limited spread, emphasizing the importance of considering RSV in the differential diagnosis of respiratory infections in healthcare settings.

Respiratory syncytial virus (RSV) is a leading cause of lower respiratory tract infection in children worldwide. In the United States, RSV causes an estimated 57 000 hospitalizations in children < 5 years of age each year [1]. Besides young chronologic age, other risk factors for severe disease include preterm birth, chronic lung disease, and congenital heart disease [2]. Outbreaks of RSV in healthcare settings have been reported globally over the last few decades, including in pediatric intensive care units, adult stem cell transplant units, adult hematology-oncology units, and neonatal intensive care units (NICUs) [3–10]. NICU outbreaks are of great concern because of the risk of severe disease in young infants, particularly those born prematurely, or those with congenital abnormalities or other comorbidities.

RSV is divided into 2 subgroups (A and B). RSV subgroups can be further divided into genotypes based on the hypervariable region of the RSV glycoprotein (G) gene [7, 8, 11, 12]. The G gene alone may not be enough to understand the relatedness of viruses among those infected.

In December 2017, the Louisiana Department of Health (LDH) was notified of 7 RSV infections that occurred among patients within a single NICU over an 11-day span [13]. We report on the epidemiological findings of the investigation and the use of whole genome sequencing (WGS) to understand the relatedness of the viruses found in patients.

## METHODS

### Healthcare Setting

The facility is a large (50–100 bed) level III NICU with 5 wards (A–E) that all have single-family rooms. There is no difference in the level of patient acuity across the different wards, and wards have shared staff. The catchment area of the facility includes a 30-mile radius, which includes 3 parishes. According to the facility’s visitation policy, parents are invited to stay over-night with their infant, and siblings > 2 years of age are encouraged to visit during daytime hours. All family and guests must wash hands before visitation. During the winter respiratory season (that covers RSV and influenza seasons), additional guidelines are in place that state visitors will have their temperature checked upon entrance to the unit and that any visitors with fever or reported respiratory symptoms will not be permitted into the unit.

### Epidemiologic and Infection Control Investigation

Three days after being notified of the cluster, LDH epidemiologists visited the facility to review medical records of RSV-infected infants and infection control measures. The team manually abstracted data on patient demographics, clinical presentation, interventions and medications, preexisting conditions, and microbiology results. The team examined infection control policies and practices including use and availability of personal protective equipment, infection control signage, environmental cleaning, and visitation policies. This investigation was determined to be a public health response by LDH and therefore did not require institutional review board approval.

### Specimen Collection and Laboratory Investigation at Hospital

After the first patient was identified, the facility implemented enhanced surveillance for RSV. This included obtaining nasopharyngeal (NP) specimens from any NICU patient who had respiratory or other RSV-related symptoms, including cyanosis, apnea, or cough, as well as from asymptomatic infants on 2 (A and B) of 3 wards where the 7 patients resided. NP swabs were collected from all 7 patients within 2 days of symptom onset and immediately placed in commercial virus transport media and frozen at −70 °C.

Specimens from the 7 NICU patients were tested initially at the hospital laboratory using a commercial respiratory virus panel. These specimens and 23 RSV specimens collected during routine surveillance between October 2017 and January 2018 from patients located in 2 parishes of Louisiana—1 where the outbreak occurred and 1 in a parish neighboring the facility catchment area (we will refer to these routinely collected specimens as “community specimens”)—were then sent to the Centers for Disease Control and Prevention’s (CDC) Respiratory Viruses Branch laboratory for further characterization [14].

### Laboratory Testing and Virus Sequencing at CDC

Hospital and all community specimens were tested using validated CDC molecular diagnostic assays to determine RSV subgroup [15]. Total nucleic acid was extracted from specimens using a magnetic silica-based platform (NucliSens easyMAG, bioMérieux, Durham, North Carolina). Nucleic acid extracts were then tested by an established pan-RSV real-time reverse-transcription polymerase chain reaction (rRT-PCR) assay [16] and a duplex rRT-PCR assay for RSV subgroup identification (A or B) [15]. WGS was performed on a subset using a 20-amplicon nested RT-PCR next-generation sequencing (NGS) method (details available upon request). In brief, RSV subgroup–specific primers were used to amplify the RSV genomes in overlapping amplicons. PCR products from each sample were pooled, purified, and used for library construction using Nextera XT DNA Sample Prep Kit (Illumina, San Diego, California). Paired-end sequencing was performed on the Ilumina MiSeq using 500-cycle MiSeq Reagent Nano Kit V2 (Illumina). A tailored NGS bioinformatics pipeline (vPipe) was used to perform read quality control and de novo assembly [17]. Sequences obtained from NICU patients and community specimens with the same RSV B genotype (including 2 specimens with RSV A co-detections) were aligned, and phylogenetic trees were computed using Bayesian analysis (Mr. Bayes version 3.2.6) and compared to 2 RSV B reference genomes (GenBank accession numbers KY924878 and KY249659). Additionally, signature nucleotides were identified by single-nucleotide polymorphism (SNP) analysis.

## RESULTS

### Patient Identification and Characteristics of RSV-Infected Infants

**Figure 1.**
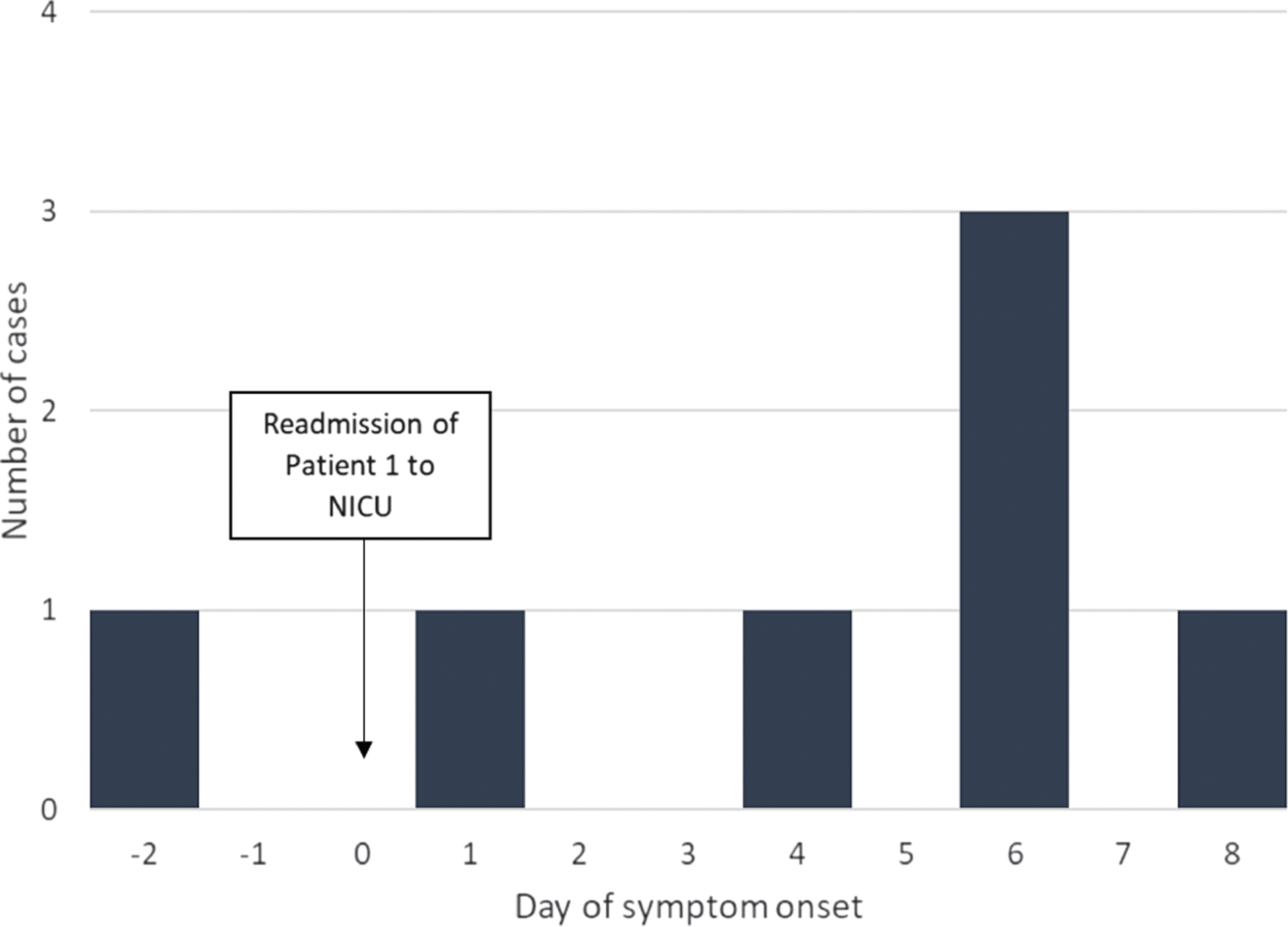
Timeline of onset of symptoms of 7 neonatal intensive care unit (NICU) patients (N = 7) relative to the hospitalization of the first reported infected patient, defined as day 0.

**Figure 2.**
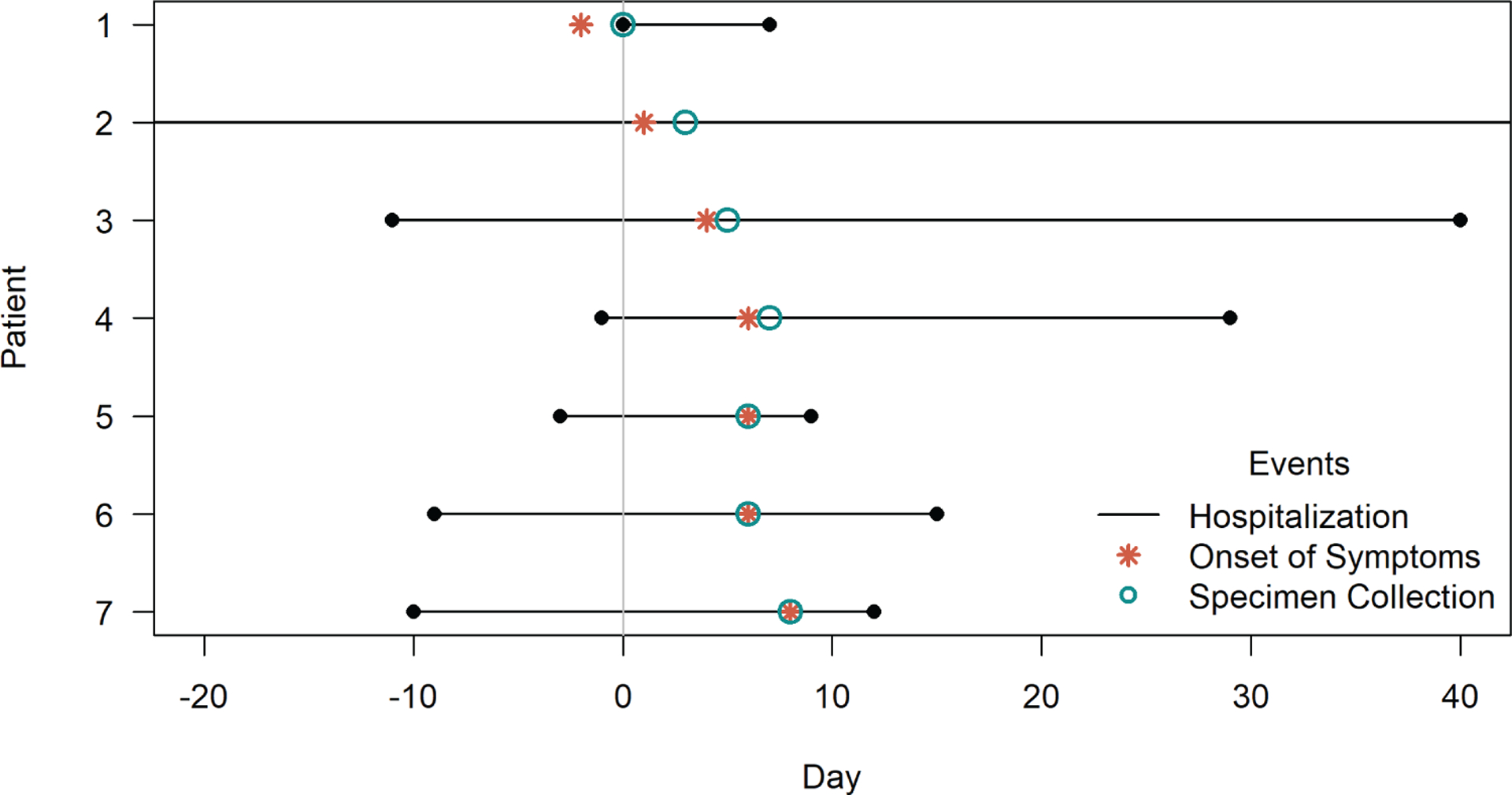
Timeline of hospitalization duration (day of admission, or day of birth for all patients except patient 1, to day of discharge), onset of symptoms, and day of specimen collection. Patient 2 was hospitalized for 272 days (day −146 to day 126); the entire hospitalization is not shown in the figure.

Characteristics of patients with RSV are shown in Table 1. Patient 1 was admitted to the NICU at 34 days of age for acute respiratory distress (day 0; Figure 1). This patient had first developed respiratory symptoms at home 2 days earlier (day −2). Patient 1 was born at the facility and had been discharged 30 days prior to readmission. RSV infection was confirmed by rRT-PCR at the facility’s laboratory upon admission on day 0. Between days 1 and 8, an additional 6 patients developed respiratory symptoms and were diagnosed with RSV at the facility laboratory (Table 1, Figure 2). On day 11, the facility notified LDH of the cluster.

**Figure 3.**
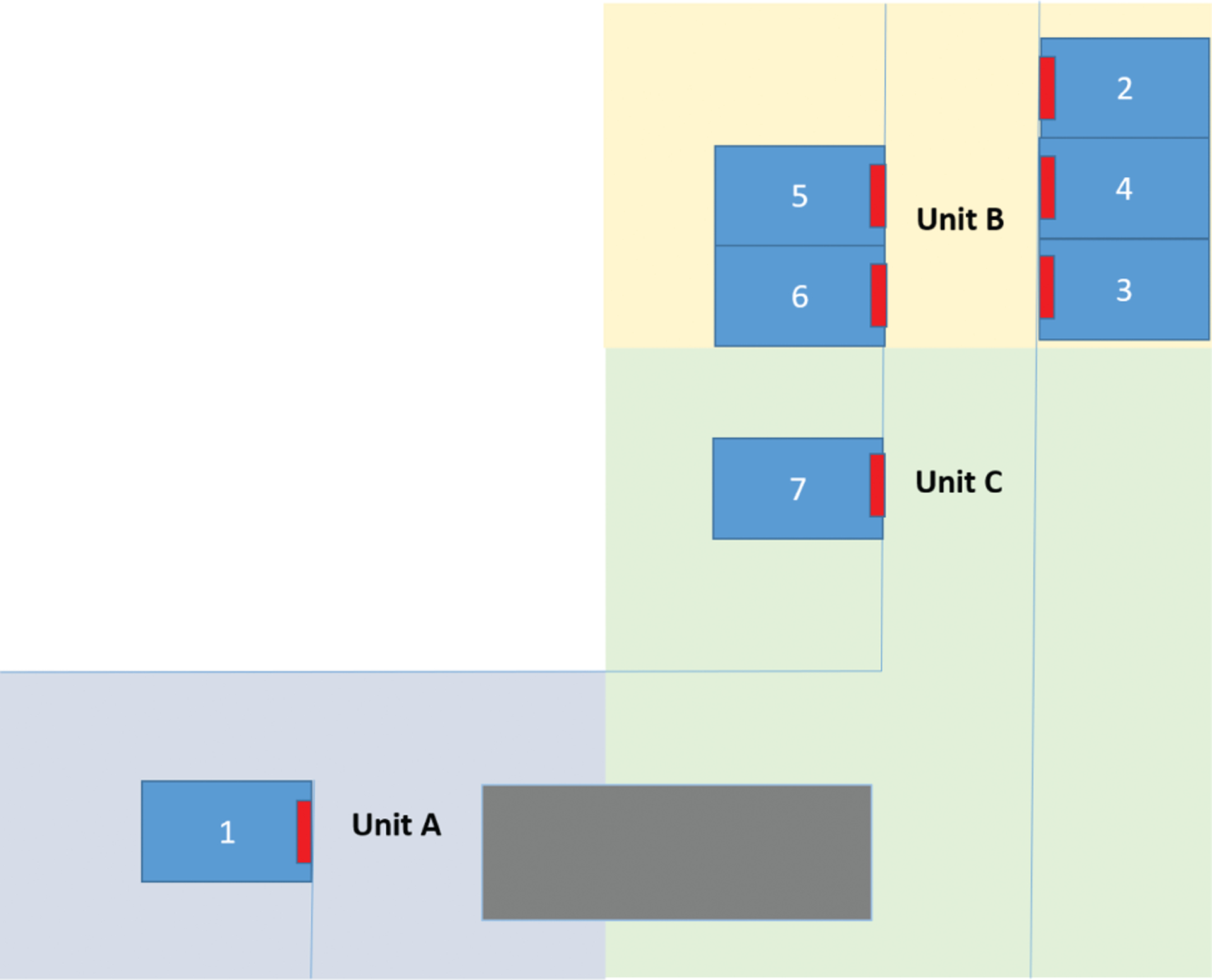
Unit and room locations of the 7 respiratory syncytial virus–infected patients. Blue boxes indicate patient rooms, red boxes indicate patient room doors, and open blue lines indicate hallways between units and rooms. The grey box indicates a storage room.

No additional RSV infections were identified after the 7 patients originally reported. All the patients were born at the facility, and no patients were on respiratory support prior to onset of symptoms. Other than the first patient, no patients had ever left the NICU before onset of symptoms. Six patients (86%) were preterm with gestational ages at birth that ranged from 25 to 36 weeks (median gestational age, 34 weeks; Table 1). One patient had chronic lung disease. Median chronological age at symptom onset was 15 days (range, 7–147 days; Table 1). One patient was located on ward A, 5 on ward B, and 1 on ward C (Table 1, Figure 3). The 6 patients located on wards B and C were all located in adjacent single-family rooms (Figure 3).

### Clinical Course and Outcomes

Patients most commonly presented with congestion (n = 7 [100.0%]), cough (n = 4 [57.1%]), tachypnea (n = 4 [57.1%]), and poor feeding (n = 4 [57.1%]) (Table 2). Three patients had abnormal chest radiographs, including 2 with acute signs of lower respiratory disease: 1 with left lower lobe infiltrates, and 1 with bilateral infiltrates. Three patients required additional respiratory support after onset of symptoms (Table 1): 1 required bilevel positive airway pressure (patient 2), 1 required high-flow nasal cannula (patient 4), and 1 required continuous positive airway pressure and was intubated 5 days after symptom onset (patient 3). Four patients required intravenous fluids, and 1 received antibiotics. No patients received palivizumab either before or after becoming ill. Median length of hospitalization from symptom onset to hospital discharge was 9 days (range, 3–125 days; Table 1). All patients recovered and were discharged from the facility.

### Laboratory Results

At the hospital laboratory, all patient specimens were identified as RSV B and were negative for influenza, coronavirus, parain-fluenza, and human metapneumovirus by rRT-PCR. Patient 2 had a co-detection of RSV with rhinovirus/enterovirus by rRT-PCR at the hospital laboratory (Table 1). CDC retested the 7 NICU patients and 22 community controls using RSV pan and duplex rRT-PCR assays. All 7 specimens from NICU patients were identified as RSV B. Of the 22 community specimens, 14 were identified as RSV B, 6 were identified as RSV A, and 2 were identified as A and B co-detections.

Except for 1 community specimen with very low viral load (cycle threshold value, 37.2), complete genome sequences were obtained from 28 specimens. Average coverage was 99 times to 171 times and genome coverage exceeded 99.1% for all samples tested. Nucleotide alignment of the obtained G gene sequences revealed that all RSV B strains were genotype BA and all RSV A strains were genotype ON1.

**Figure 4.**
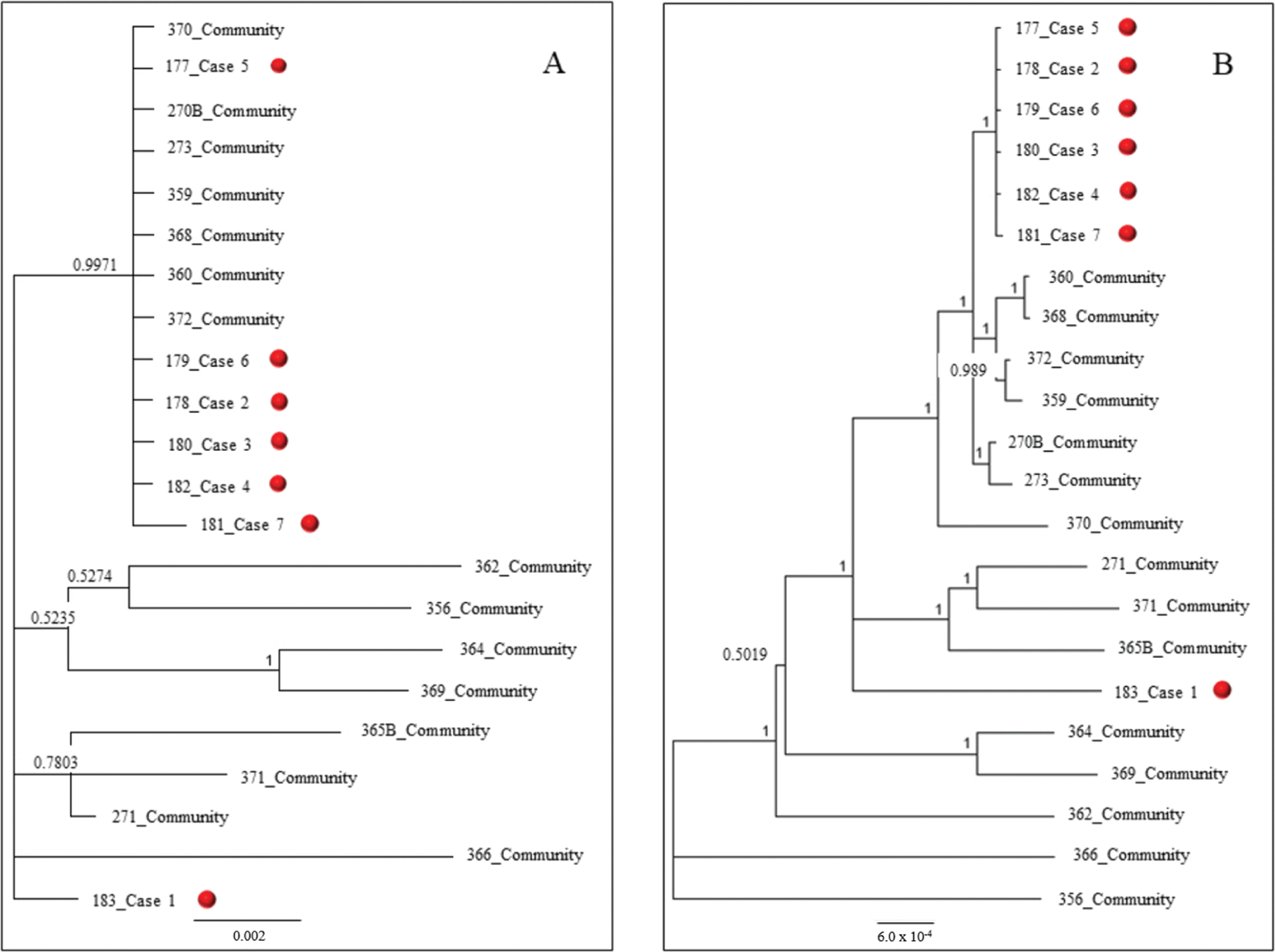
Phylogenetic trees of respiratory syncytial virus (RSV) G gene (*A*) and whole genome sequencing (WGS; *B*) from community and cluster sequences using Bayesian analysis (Mr. Bayes version 3.2.6), applying general time reversible (GTR) substitution model and gamma rate variation. The RSV G gene and WGS from the patients are highlighted with red dots. Sequences from this study include GenBank accession numbers MJ929516-MJ929538.

Phylogenetic analysis of the G gene alone segregated patient 1 and patients 2–7 into 2 clusters (Figure 4A), with patients 2–7 clustering with some community patients. Phylogenetic analysis of the RSV WGS further segregated NICU patients 2–7 and community patients into unique clusters (Figure 4B). NICU patients 2–6 were 100% identical, and patient 7 had 99.99% nucleotide identity by pairwise uncorrected comparison to patients 2–6. By WGS, patient 1 shared 99.5% nucleotide identity with patients 2–7, and the community specimens had 99.16%–100.00% nucleotide identity. In contrast, NICU and community specimens shared 97.60%–98.23% (KY924878) and 96.03%–96.97% (KY249658) nucleotide identity when compared to reference strains of RSV B.

SNP analysis identified 3 unique signature nucleotides in patients 2–7 that were absent in patient 1 and the community specimens; 2 unique SNPs were found in the M2–1 gene and 1 in the polymerase (L) gene. The unique SNPs and nucleotide agreement among viruses found in patients 2–7 suggest a single introduction of RSV into the NICU that resulted in infections among those patients, and distinguish that introduction from that of patient 1, which did not result in additional infections.

### Infection Control

After identification of the first patient, the NICU implemented contact precautions, in addition to standard precautions, for all symptomatic infants. All NICU healthcare workers were asked to self-report any respiratory symptoms at the start of each shift. Additionally, all parents of NICU patients were sent a letter outlining guidelines issued during the winter respiratory season that included routine screening of visitors for fever and disallowing visitation of anyone with respiratory symptoms. LDH, in consultation with CDC, recommended additional precautions to limit transmission, including restriction of visitation from children < 12 years of age, use of surgical face masks during visitation by nonstaff, increased hand hygiene stations, enhanced environmental cleaning, and cohorting of staff. In the context of this cluster, droplet precautions were also recommended and implemented by the facility. Notices were placed outside the rooms of patients, as were signs describing appropriate contact and droplet precautions for visitors and staff.

The staff reported that the mother and siblings of the first patient (patient 1) had respiratory symptoms at the time of their NICU readmission. In addition, NICU staff observed that 2 patients were visited by family members with respiratory illnesses prior to the implementation of enhanced infection control measurements, but the specific timing of those visits relative to the patients’ onset of symptoms was not known. No NICU healthcare workers reported any symptoms and none were tested for RSV.

## DISCUSSION

To our knowledge, this is the first study to use WGS to understand the relatedness of viruses in an RSV outbreak within a NICU. Combining sequencing and epidemiologic data, we found that RSV infections among patients were likely caused by 2 distinct introductions of the virus into the NICU. One introduction resulted from RSV being brought into the unit through readmission of a symptomatic infant who had been previously discharged. There was no evidence that this introduction led to other illnesses among NICU patients. The other introduction likely came from the community through a visitor or staff and resulted in 6 additional RSV infections among NICU patients. Despite awareness of the first 2 infections, RSV likely spread to additional patients through shared staff and/or equipment. After implementation of strict infection control measures and enhanced surveillance, no further patients were identified among NICU patients, and all recovered from their infections. RSV testing was not routine prior to recognition of the outbreak, but surveillance screening was instituted among all patients, symptomatic or not, in the affected wards and among all patients with respiratory symptoms throughout the facility. Rapid identification and awareness of the infections as well as effective infection control practices were critical to limiting transmission of RSV.

RSV outbreaks have been reported in NICUs and other healthcare settings that care for newborns [3–5, 10, 12, 18–26]. Among publications describing nosocomial transmission of RSV globally since 2000 in newborn inpatient settings, outbreaks ranged from 2 patients in a NICU in Austria to 23 patients during a 3-month time period in South Africa [12, 23]. In 10 of the outbreaks described, palivizumab was given to at least 1 infant in order to contain transmission [3, 4, 10, 18–20, 22–24, 26]. The WGS results in our investigation led to the conclusion that the outbreak described here was likely caused by 2 separate introductions within a short time span.

The first patient diagnosed with RSV developed symptoms after having been discharged > 30 days prior to readmission to the NICU. Since the incubation period for RSV is 2–8 days, this patient’s exposure likely occurred outside the NICU [2]. Analysis of WGS identified an RSV viral genome from the first patient that was distinct from viruses from the other 6 patients, who shared nearly identical virus genomes and 3 unique SNPs. These 6 infants never left the facility postpartum, developed symptoms within a 7-day period, and were located in adjacent rooms (Figure 3). This evidence suggests that a second, distinct introduction of RSV occurred in the NICU and that the virus spread among these 6 patients. Although the G gene sequence could distinguish the strain from patient 1 compared to patients 2–6, it could not discriminate between outbreak and nonoutbreak strains. WGS was able to distinguish outbreak from nonoutbreak strains, highlighting that infections in patients 2–6 were likely caused by a single introduction. Our findings, along with those from previous studies, suggest that WGS is necessary for understanding patterns of RSV transmission that occur over short time periods [27, 28].

During months of high community RSV circulation (typically fall through spring in the United States), healthcare facilities are at increased risk for introduction of RSV [29]. In Louisiana, RSV circulation peaked in mid-November during the 2017–2018 season, approximately 2 weeks before the cluster was observed. This NICU has a policy of allowing readmission of patients previously discharged to the community. Some NICUs are “closed” to infants previously discharged to prevent spread of infections. Despite a NICU policy banning ill visitors, staff reported that 2 patients were visited by family members with respiratory symptoms. In addition, the facility did not have a policy limiting visitation in the NICU by young children, who are the most common transmitters of RSV [30–32]. While recognition of ill visitors and staff may prevent the spread of RSV, RSV can also be transmitted by asymptomatic persons [33].

Transmission of RSV within the NICU was likely limited because of early recognition and enhanced surveillance. Infants in the NICU are often evaluated for “late-onset sepsis” but it is unclear how often viral respiratory pathogens are suspected.” Recent studies have found that among patients evaluated for late-onset sepsis, 7%–10% had respiratory viruses detected, and among these, RSV was a common respiratory virus isolated [34–36]. Awareness and early detection of RSV could prevent outbreaks within the NICU and prevent unnecessary use of antibiotics.

To prevent RSV transmissions in healthcare settings, infection control measures are often multifaceted. In addition to standard and contact precautions, such measures may include patient and staff cohorting, as well as patient and staff screening [33, 37–41]. The additional benefit of using droplet precautions, which were implemented during this outbreak, remains unclear. Although palivizumab is often used in healthcare settings to control outbreaks of RSV, the American Academy of Pediatrics does not recommend its use for this purpose, but rather recommends adherence to strict infection control policies [2, 3, 10, 19, 22, 26]. There is a lack of evidence that palivizumab reduces transmission of RSV or improves clinical outcomes in nosocomial outbreaks, and in this situation it was not given to any at-risk patients in the facility. A recent review of nosocomial transmission of RSV suggested a lack of evidence regarding which control strategies are most effective and cost-effective at reducing transmission in healthcare facilities [38]. Studies evaluating the most effective infection control measures would be valuable.

## CONCLUSIONS

Policies that ban visitation by persons with respiratory symptoms and all young children when RSV is circulating may help prevent introduction of RSV into healthcare settings. This cluster also highlights the importance of early recognition of RSV, which should be considered when evaluating NICU patients for respiratory symptoms or sepsis, particularly during RSV season.

## Figures and Tables

**Table 1.** Epidemiological and Clinical Characteristics of Patients (N = 7)

Patient Number	Gestational Age at Birth, wk	Age at Onset, d	Day of Onset of Symptoms^a^	Unit	Length of Hospitalization^b^, d	Comorbidities	Diagnosis	Respiratory support^c^
1	37	31	−2	A	7	None	RSV B	No
2	25	147	1	B	125	Chronic lung disease; premature	RSV B, rhinovirus/enterovirus	BiPAP
3	34	15	4	B	36	Jaundice; hemorrhagic disease	RSV B	CPAP; intubated
4	36	7	6	B	23	None	RSV B	High-flow cannula
5	34	9	6	B	3	None	RSV B	No
6	34	15	6	B	9	None	RSV B	No
7	36	18	8	C	4	None	RSV B	No

Abbreviations: BiPAP, bilevel positive airway pressure; CPAP, continuous positive airway pressure; RSV, respiratory syncytial virus.

a Relative to the hospitalization of the first identified case (day 0).

b From symptom onset to discharge; length of stay not necessarily due to RSV infection.

c Additional respiratory support required after symptom onset.

**Table 2.** Description of Observed Case Symptoms (N = 7)

Symptom	Count (%)
Congestion	7 (100)
Cough	4 (57)
Poor feeding	4 (57)
Tachypnea	4 (57)
Dyspnea	2 (29)
Lethargy	1 (14)
Wheezing	1 (14)
Fever	0 (0)
